# ViroBioTree: A Tree-Structured Biological Evidence Retrieval Framework for Viral Protein Function Annotation

**DOI:** 10.3390/v18060656

**Published:** 2026-06-09

**Authors:** Tinglian Lai, Fuguo Liu, Guodong Li, Liyan Hua

**Affiliations:** 1 School of Mathematics and Computational Science, Guilin University of Electronic Technology, Guilin 541004, China; 2 School of Mathematics and Data Science, Changji University, Changji 831100, China; 3 Guangxi Colleges and Universities Key Laboratory of Data Analysis and Computation, School of Mathematics and Computing Science, Guilin University of Electronic Technology, Guilin 541002, China; 4 Center for Applied Mathematics of Guangxi, Guilin University of Electronic Technology, Guilin 541002, China; 5 Academy of Excellent Engineers, Guangxi Artificial Intelligence College, Nanning 530201, China

**Keywords:** viral protein function annotation, evidence retrieval, biological tree index, retrieval-augmented generation, evidence verification, SARS-CoV-2, Influenza A Virus, cross-family diagnostic audit, wastewater metagenomics, traceable annotation

## Abstract

Accurate viral protein function annotation is essential for genomic surveillance, yet conventional retrieval-augmented generation (RAG) pipelines often fragment biological evidence into fixed-length text chunks, disrupting relationships among ORFs, annotations, structural domains, sequence motifs, residue mappings, and model-derived attention evidence. We propose ViroBioTree, a tree-structured biological evidence retrieval framework for downstream viral protein evidence review rather than a new primary annotation classifier. Built as an evidence organization layer on ViralMultiNet-derived ORF-level predictions and annotations, ViroBioTree converts sequence, annotation, structure, and attention evidence into typed biological nodes and traceable edges, then performs deterministic multi-channel recall, evidence-aware reranking, balanced TopK selection, rule-based verification, and node-cited report generation. In a demo benchmark, ViroBioTree achieved its strongest deterministic proxy performance on structure-explanation tasks, with Precision@K = 1.0, Recall@K = 1.0, and diversity = 0.52; these values reflect expected node-type and tag agreement rather than independent biological correctness. A bounded full-scale SARS-CoV-2 index contained 39,800 ORF rows, 80,000 attention records, 199,418 nodes, and 495,886 edges. In a stratified full20k diagnostic evaluation, ViroBioTree showed task-dependent advantages over LlamaIndex vector retrieval for conflict detection, evidence retrieval, and structure explanation, while LlamaIndex remained competitive or stronger for annotation-rich function annotation. A cross-family Influenza A Virus (IAV) diagnostic audit showed that the schema can represent IAV evidence namespaces while explicitly exposing missing formal ORF inputs, missing attention evidence, and unavailable residue/PDB assertions. Supplementary robustness, external sanity-check, diversity-risk, expert-evaluation, domain-tool positioning, and cross-family audit analyses supported traceability, report quality, and conservative evidence handling, but also showed that stable Precision@K under query perturbation does not necessarily imply stable retrieved evidence sets. ViroBioTree operates offline and deterministically, but does not address raw-read assembly, base calling, primary ORF prediction, or wet-lab validation. Its results should be interpreted as proxy and expert-reviewed evidence for traceable viral protein evidence retrieval and report generation rather than as direct validation of biological function annotation.

## 1. Introduction

Accurate functional annotation of viral open reading frames (ORFs) is central to genomic surveillance, variant risk assessment, and pandemic preparedness [1,2]. Determining whether an ORF encodes an enzymatic protein (e.g., RNA-dependent RNA polymerase), a structural protein (e.g., Spike glycoprotein), a transport protein (e.g., viroporin), or a regulatory accessory factor directly informs assessments of viral fitness, host-range determinants, and drug target accessibility [3]. The emergence of wastewater-based epidemiology as a frontline surveillance modality has intensified the need for automated, interpretable annotation tools capable of processing fragmented metagenomic sequences at scale.

Large language models (LLMs) and retrieval-augmented generation (RAG) systems offer a route for combining biological text, database evidence, structural information, and model outputs [4,5]. However, standard RAG pipelines decompose knowledge corpora into fixed-length text chunks, retrieve the top-K most semantically similar chunks via dense vector search [6], and condition LLM generation on the retrieved context. This architecture exhibits three fundamental limitations when applied to viral protein annotation.

Fixed chunking can break biological evidence boundaries [7,8]. For example, receptor-binding domains (RBD, residues 319–541), cleavage sites (residues 681–685), catalytic motifs, residue ranges, and high-attention k-mers may be split across unrelated chunks, severing the structural continuity required for mechanistic interpretation. Standard vector retrieval is also evidence-level agnostic: it cannot distinguish whether a retrieved chunk corresponds to ORF-level sequence data, protein-level functional annotation, or model-derived attention evidence. Finally, without structured verification, LLMs are prone to generating unsupported biological claims [9].

**Figure 1:**
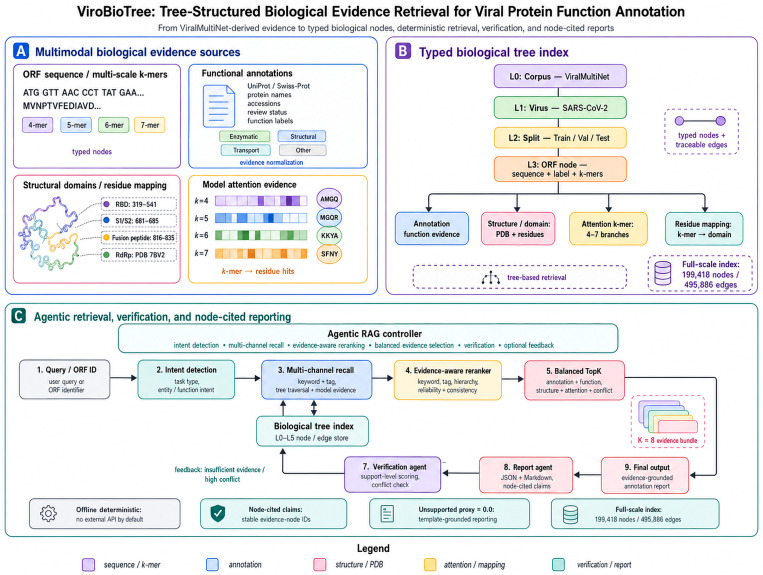
Overall architecture of the ViroBioTree framework. ViroBioTree integrates ViralMultiNet-derived multimodal biological evidence, including ORF sequences, multi-scale k-mers, functional annotations, structural domains, residue mappings, and model attention evidence. These heterogeneous evidence sources are normalized into typed biological nodes and traceable edges to construct a tree-structured biological index. Given a query or ORF identifier, the agentic retrieval controller performs intent detection, multi-channel recall, evidence-aware reranking, balanced TopK evidence selection, verification, and node-cited report generation. The framework operates offline and deterministically by default, producing evidence-grounded reports with explicit evidence-node citations.

ViroBioTree addresses this gap by replacing fixed text chunks with a biological tree-structured evidence index (Figure 1). The proposed system treats sequence records, annotation fields, domain definitions, residue mappings, and attention k-mers as typed nodes connected by explicit edges. Retrieval is performed over these biological nodes rather than unstructured text chunks. A deterministic reranking and evidence-balanced TopK module then compresses wide recall results into compact, traceable evidence bundles. Finally, a conservative rule-based Verification Agent and template-based Report Agent generate evidence-grounded reports without requiring a live LLM API.

In this manuscript, annotation refers to structured protein-level functional and evidence metadata rather than de novo database curation. This includes UniProt/Swiss-Prot protein names, accessions, functional descriptions, review status, ViralMultiNet functional class labels, and linked domain, motif, residue, and model-attention evidence. ViroBioTree retrieves, organizes, and reports these annotations as traceable evidence bundles; it does not replace UniProt or other experimentally curated annotation databases.

The work is positioned as a systems and methods contribution rather than a new viral classifier. ViralMultiNet [3] is used as a source of prediction and interpretability evidence, while ViroBioTree focuses on organizing, retrieving, verifying, and reporting that evidence in a traceable manner. This separation ensures that all functional predictions originate from a previously reported upstream deep learning model [3,10] rather than from LLM inference.

### Main Contributions

1. We propose a biological tree-structured index that organizes viral annotation evidence across corpus, virus, ORF, function, structural domain, and motif/residue/attention-evidence levels.
2. We implement multi-channel candidate recall and evidence-aware reranking over typed biological nodes, using keyword, tag, hierarchy, reliability, prediction-consistency, and diversity signals.
3. We introduce evidence-balanced top-K selection to preserve complementary annotation, functional, structural, attention, and conflict evidence in compact retrieved bundles.
4. We develop an offline rule-based Verification Agent and template-based Report Agent that generate node-cited viral protein evidence reports without requiring an external LLM API.
5. We assess scalability using a bounded full-scale SARS-CoV-2 index comprising 39,800 ORF rows, 80,000 attention records, 199,418 nodes, and 495,886 edges, and further report a stratified full20k sampled evaluation that improves structure/attention evidence coverage and produces distinguishable reduced-ablation effects under deterministic proxy metrics.
6. We evaluate cross-family diagnostic portability with an Influenza A Virus (IAV; Orthomyxoviridae) evidence-availability audit, testing whether the same typed hierarchy can represent another viral family while explicitly reporting missing formal ORF inputs, attention evidence, and residue/PDB assertions.

## 2. Related Work

### 2.1. Viral Protein Function Annotation

Computational viral protein annotation has progressed from homology-based sequence search and profile matching toward deep learning and multimodal approaches. Alignment tools such as DIAMOND (version 2.1.8) [11] and BLAST (version 2.15.0) [12] provide high precision on well-characterized viruses but degrade on novel or highly mutated sequences where reference coverage is sparse. Deep learning models, including HyenaDNA [13] and GenSLMs [14], extract hierarchical features directly from nucleotide sequences using transformer-based architectures [10], demonstrating strong benchmark performance but limited sensitivity when functional motifs are obscured by mutations. Language model approaches such as BioBERT (version 1.1) [15,16] fine-tuned on UniProt [17] functional descriptions capture rich semantic information but operate independently of genomic sequence context, reducing effectiveness when annotation coverage is incomplete. A prior multimodal framework, ViralMultiNet, integrates multi-scale k-mer encodings (k = 4–7) with UniProt annotation embeddings under gated multimodal fusion and triple knowledge distillation [18], achieving strong classification performance on a 66,011-sample SARS-CoV-2 wastewater dataset. ViroBioTree addresses the gap in interpretability by building a traceable evidence retrieval and verification framework on top of ViralMultiNet predictions, using sequence handling utilities from established bioinformatics toolkits [19].

### 2.2. Retrieval-Augmented Generation

Retrieval-augmented generation (RAG) augments generative models with externally retrieved context to improve factual grounding and reduce hallucination [4]. Standard RAG pipelines decompose reference documents into fixed-length chunks, encode them as dense vectors, and retrieve the top-K chunks by cosine similarity before conditioning LLM generation. Advanced variants address limitations of fixed chunking: parent–child RAG retrieves fine-grained child chunks but returns broader parent context to preserve coherence; semantic chunking segments documents at natural linguistic boundaries; and HyDE [20] uses hypothetical document embeddings to improve recall for rare queries. Survey work on RAG for LLMs [6] identifies evidence complementarity and source reliability as open challenges that existing retrieval frameworks do not address. REPLUG [21] and related black-box RAG approaches improve factual grounding in general-domain tasks but do not incorporate domain-specific biological evidence hierarchy.

### 2.3. Agentic RAG and Tree-Structured Indexing

Agentic RAG extends retrieval with planning, multi-step tool use, and verification [22,23]. Frameworks such as ReAct [24] enable LLMs to iteratively generate reasoning traces and execute tool calls, improving performance on knowledge-intensive tasks. Toolformer [25] demonstrates that LLMs can learn to invoke external APIs, including search engines and calculators, in a self-supervised manner. LlamaIndex [22] introduces hierarchical document indexing with parent–child node relationships, improving retrieval coherence for structured documents. Microsoft GraphRAG [23] represents knowledge as a graph and performs community detection to support multi-hop reasoning across large corpora. However, these frameworks are designed for general-domain text corpora and do not incorporate domain-specific biological semantics. ViroBioTree differs by constructing a biologically motivated six-level hierarchy with typed evidence nodes and tag-based retrieval explicitly designed for the hierarchical structure of viral functional knowledge, where ORF-to-protein-to-domain-to-residue relationships carry biological meaning that general-purpose document trees cannot represent.

### 2.4. LLMs in Bioinformatics

LLMs have been explored for biomedical question answering, gene and protein annotation, and workflow automation. BioGPT [26] is a domain-specific generative transformer pre-trained on PubMed literature, achieving state-of-the-art results on biomedical relation extraction and question answering. GeneGPT [27] demonstrates that LLMs can be augmented with NCBI [28] Web API calls to answer genomics questions that require precise database lookups, reducing hallucination through tool-grounded generation. GPT-based models [5] have also been applied to medical licensing examinations [29], highlighting both their capabilities and their tendency to hallucinate when precise domain-specific claims are required. ViroBioTree addresses this challenge specifically for viral protein annotation by separating the prediction step (ViralMultiNet) from the evidence reasoning step (ViroBioTree), and by using a rule-based Verification Agent that explicitly quantifies evidence support levels and flags unsupported claims before report generation.

## 3. Materials and Methods

### 3.1. Overview of ViroBioTree

**Figure 2:**
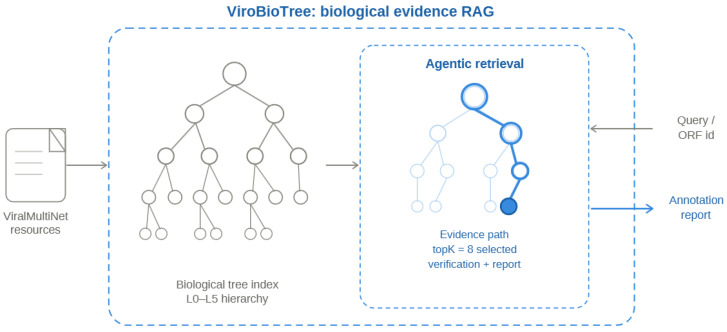
Overview of the ViroBioTree framework. The framework converts ViralMultiNet-derived sequence, annotation, structure, and attention evidence into a biological tree-structured index, then performs deterministic retrieval, evidence-aware reranking, balanced evidence selection, verification, and report generation.

ViroBioTree is a biological tree-structured retrieval framework designed to organize curated and model-derived virological evidence for traceable downstream reporting (Figure 2). In contrast to conventional RAG pipelines that decompose knowledge into fixed-length chunks, ViroBioTree organizes evidence into a six-level biological hierarchy, preserving ORF-to-domain-to-residue relationships. The framework operates in three phases. Phase 1 constructs a biological tree index from ViralMultiNet dataset resources, including ORF sequences, UniProt/Swiss-Prot [17] annotations, structural domain definitions derived from experimentally resolved SARS-CoV-2 protein structures (Spike PDB 6VXX; RdRp PDB 7BV2), and multi-scale attention k-mer evidence from ViralMultiNet’s four parallel attention branches (k = 4, 5, 6, 7). Phase 2 performs multi-channel candidate recall, evidence-aware reranking, and evidence-balanced top-K selection to construct a compact, complementary evidence bundle. Phase 3 applies a rule-based Verification Agent and template-based Report Agent to generate node-cited annotation reports without requiring an external LLM API call. The default implementation is fully offline and deterministic, ensuring full reproducibility independent of API availability.

### 3.2. Proteome-Scale Application Workflow

For a virus of interest, ViroBioTree can be instantiated at the proteome scale by creating one virus node and one ORF/protein node per protein record, then attaching available annotation, domain or motif, residue, and model-evidence nodes. The required inputs are an ORF/protein table, functional labels or homology-derived annotations, optional curated domain/motif definitions, optional residue or structure mappings, and optional model-attention or other interpretability evidence. If a domain or motif source is supplied, ViroBioTree can retrieve and report that information as explicit biological evidence nodes; if such evidence is absent, the Verification Agent reports missing support rather than fabricating motif or domain claims.

A step-wise RNA-virus example is the IAV proteome workflow used here as a diagnostic template. First, collect protein records for HA, NA, M1, M2, NP, PB1, PB2, PA, NS1, and NS2. Second, import UniProt/Swiss-Prot or homology-derived protein annotations and review-status metadata. Third, attach available motif and domain evidence, such as HA receptor-binding, cleavage, fusion, stem/head regions, NA catalytic evidence, M2 transmembrane or ion-channel evidence, polymerase-domain evidence, and NP RNA-binding evidence. Fourth, construct the L0–L5 hierarchy linking the virus, proteins, annotations, domains or motifs, residues, and model-evidence nodes. Fifth, run a multi-channel recall for a query such as “What evidence supports HA receptor-binding domain annotation?” Sixth, apply evidence-balanced TopK selection so that annotation, domain, structural, attention, and conflict evidence are represented when available. Finally, the Verification Agent assigns support levels and the Report Agent emits node-cited evidence summaries. The current IAV analysis remains an evidence-availability audit because formal local IAV ORF/protein rows and attention records were unavailable; with those inputs supplied, the same workflow can be applied to a specific virus proteome.

### 3.3. Biological Tree-Structured Index Construction

**Figure 3:**
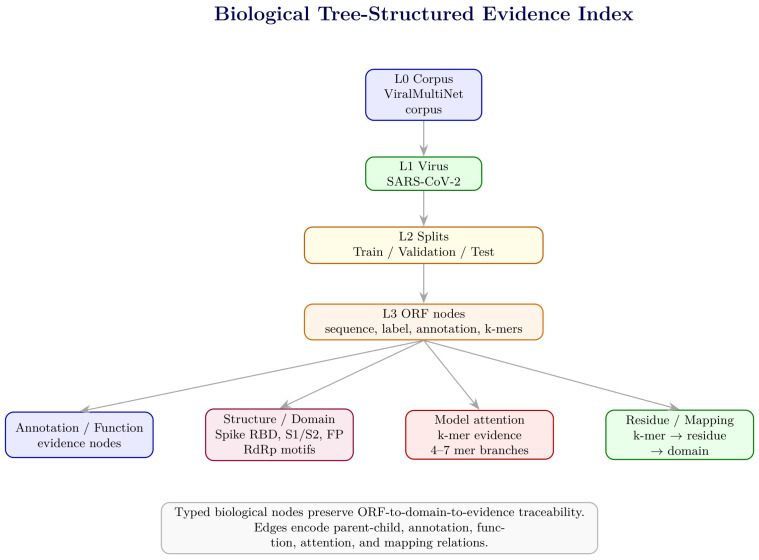
Biological tree-structured index. ViroBioTree organizes the corpus into a SARS-CoV-2 root, train/validation/test split nodes, ORF nodes, annotation/function evidence, structure/domain namespaces, and high-attention k-mer evidence. This structure preserves the biological context that is lost in fixed chunk retrieval.

The index schema is rooted at a corpus node (L0) and a virus-specific node (L1), allowing the same hierarchy to instantiate a SARS-CoV-2 index for the primary evaluation and an IAV evidence-availability audit for cross-family diagnostic portability. In the SARS-CoV-2 setting, split nodes under the L1 virus node represent train, validation, and test subsets containing 20,000, 9900, and 9900 ORF rows respectively. Each ORF node (L2) stores the nucleotide sequence, functional label (0 = Enzymatic, 1 = Structural, 2 = Transport, 3 = Other), sequence length, multi-scale k-mer tokenizations (text_4mer through text_7mer), and a reference to the original dataset row. Annotation nodes (L3) store the full UniProt/Swiss-Prot functional annotation text associated with each ORF, including protein name, accession numbers, review status, and functional description fields; nodes derived from manually reviewed Swiss-Prot records receive the reliability tag experimentally_reviewed. Structural domain nodes (L4) encode experimentally validated functional regions: Spike RBD (residues 319–541, PDB 6VXX), S1/S2 cleavage site (residues 681–685), fusion peptide (residues 816–835), and RdRp catalytic domain (PDB 7BV2 [8]). Attention k-mer evidence nodes (L5) are populated from the four high-attention k-mer FASTA files produced by ViralMultiNet, one per branch, encoding the k-mer sequence, position within the ORF, attention score derived from transformer self-attention [10], and the number of hits in the reference protein sequence from kmer2res mapping files. In the IAV audit setting, the same L0–L5 schema is retained as a template, but formal ORF/protein rows and SARS-CoV-2-specific attention nodes are not fabricated when unavailable. Nodes with reference hits receive evidence tag structure_mapping, providing a direct link between model attention and experimental structural knowledge (Figure 3).

### 3.4. Node and Edge Schema

Each node includes a stable identifier, node type, hierarchy level, parent identifier, child identifiers, optional text content, and a structured tag schema comprising four tag categories. Entity tags identify the biological entity type: corpus, virus, ORF, protein, domain, motif, or residue. Function tags encode the functional category association derived from ground-truth labels: enzymatic, structural, transport, or other. Evidence tags describe the type of biological evidence encoded: sequence, annotation, PDB, structure, k-mer, model_attention, or structure_mapping. Reliability tags encode the epistemic confidence of the evidence: experimentally_reviewed (Swiss-Prot curated), experimentally_supported (PDB-derived domain), curated_dataset (ViralMultiNet label), model_derived (attention k-mer), or low_confidence. These tags serve as filter and scoring inputs for multi-channel recall and evidence-aware reranking, enabling the retrieval layer to distinguish high-reliability annotation evidence from model-derived interpretability signals without discarding either.

### 3.5. Multi-Channel Candidate Recall

**Figure 4:**
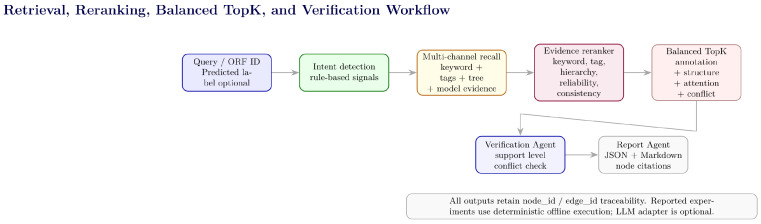
Retrieval, reranking, balanced TopK, and verification workflow. A query or ORF identifier is converted into deterministic intent signals, used for multi-channel recall, reranked by keyword, tag, hierarchy, reliability, and prediction-consistency scores, compressed into an evidence-balanced TopK bundle, and passed to a rule-based verification/reporting layer.

The retrieval layer performs deterministic multi-channel recall to construct a candidate set of up to N = 50 nodes before reranking. Keyword recall searches for node identifiers, node types, text fields, tags, and string attributes for exact and fuzzy matches against a biological vocabulary derived from the query, including protein family terms (spike, polymerase, protease, helicase, viroporin), functional domain terms (RBD, catalytic, cleavage, fusion), and modification terms. Tag recall matches query-derived intent tags to function tags, evidence tags, and reliability tags stored on each node, providing high-precision filtering for task-specific evidence types. Tree traversal recall exploits parent–child relationships in the biological tree: given a target ORF node, traversal retrieves its annotation, domain, and k-mer children (downward), its split and virus ancestors (upward), and sibling ORF nodes sharing the same functional label (lateral). Model evidence recall queries L5 attention k-mer nodes using ViralMultiNet prediction outputs as anchors, retrieving k-mer nodes from ORFs sharing the same predicted label and exhibiting high domain hit counts. The four channels are summarized in Figure 4; the unified candidate set is obtained as

(1) $$C=C_{\mathrm{keyword}}\cup C_{\mathrm{tag}}\cup C_{\mathrm{tree}}\cup C_{\mathrm{model}},$$

where $C_{\mathrm{keyword}}$, $C_{\mathrm{tag}}$, $C_{\mathrm{tree}}$, and $C_{\mathrm{model}}$ denote the candidate sets retrieved by keyword recall, tag recall, tree traversal recall, and model evidence recall, respectively. Duplicate candidates are removed by node identifier before reranking.

### 3.6. Evidence-Aware Reranking

Candidate nodes are reranked using a deterministic weighted score combining six biological relevance dimensions: $S=0.25S_{\mathrm{keyword}}+0.20S_{\mathrm{tag}}+0.20S_{\mathrm{hierarchy}}+0.15S_{\mathrm{reliability}}+0.15S_{\mathrm{consistency}}+0.05S_{\mathrm{diversity}}$. $S_{\mathrm{keyword}}$ is the normalized term frequency of query biological keywords in node text fields, drawing on established information retrieval principles. $S_{\mathrm{tag}}$ is the Jaccard similarity between query-derived intent tags and node tag fields, weighted by tag category. $S_{\mathrm{hierarchy}}$ is a level-appropriateness score: structure explanation queries score L4 domain and L5 k-mer nodes highest, while function annotation queries score L3 annotation nodes highest. $S_{\mathrm{reliability}}$ assigns tier scores after normalizing reliability tags: experimentally supported structural evidence = 1.0, Swiss-Prot reviewed annotation = 0.9, curated dataset label = 0.8, model-derived attention evidence = 0.7, predicted or unreviewed evidence = 0.5, and low-confidence evidence = 0.2. $S_{\mathrm{consistency}}$ scores 1.0 if the node function tag matches the ViralMultiNet predicted label, 0.5 if neutral, and 0.0 if contradictory. $S_{\mathrm{diversity}}$ is implemented as a low-weight diversity prior that discourages redundant evidence types without overriding reliability or hierarchy relevance. The final coefficients were selected on the validation split of the demo benchmark from a bounded grid of non-negative candidate weights in 0.05 increments, constrained to sum to one. Selection prioritized mean Recall@K and diversity on structure-explanation and evidence-retrieval tasks while preserving Precision@K and unsupported-claim proxy constraints; the selected weights were then fixed for all reported experiments.

(2) $$\begin{array}{cc} S= & w_{1}S_{\mathrm{keyword}}+w_{2}S_{\mathrm{tag}}+w_{3}S_{\mathrm{hierarchy}}+w_{4}S_{\mathrm{reliability}}\\ \; & +w_{5}S_{\mathrm{consistency}}+w_{6}S_{\mathrm{diversity}},\;\sum_{i=1}^{6}w_{i}=1. \end{array}$$

where $S_{\mathrm{keyword}}$, $S_{\mathrm{tag}}$, $S_{\mathrm{hierarchy}}$, $S_{\mathrm{reliability}}$, $S_{\mathrm{consistency}}$, and $S_{\mathrm{diversity}}$ denote keyword matching, tag matching, hierarchy relevance, reliability, prediction consistency, and diversity scores, respectively. The weights $w_{1},\ldots ,w_{6}$ are non-negative coefficients, sum to one, and are kept fixed across all reported experiments after validation selection.

### 3.7. Evidence-Balanced Top-K Selection

Instead of selecting the global top-scoring nodes, ViroBioTree applies evidence-type quotas to ensure complementary coverage. The default top-K bundle (K = 8) contains: 2 annotation evidence nodes (L3, evidence_tag ∈{annotation}); 1 function/protein node (L3, function metadata); 2 structural/domain evidence nodes (L4, evidence_tag ∈{PDB,structure}); 2 attention k-mer evidence nodes (L5, evidence_tag ∈{model_attention}); and 1 conflict or negative evidence node (any level with function tag contradicting the predicted label, or zero-hit k-mer). Within each quota category, nodes are ranked by *S* and the top-scoring nodes are selected. If a quota category cannot be filled from the candidate set, the missing evidence type is recorded and remaining slots are filled by the highest-ranked unselected nodes, preserving deterministic order by score and node identifier. Conflict evidence is triggered when function tags fall into a pre-specified contradictory pair relative to the predicted label, such as structural versus enzymatic/transport or enzymatic versus structural/transport. This evidence-balanced selection ensures that the Verification Agent and Report Agent receive an auditable evidence chain spanning annotation support, structural domain support, model attention support, and conflict assessment when those evidence types are available, while explicitly reporting missing categories when they are absent [30].

### 3.8. Verification Agent

The Phase 3 Verification Agent is rule-based by default. It assigns a support level to the ViralMultiNet predicted label by examining the retrieved evidence bundle across five dimensions: annotation match (whether annotation nodes contain keywords consistent with the predicted function), structure/domain match (whether retrieved domain nodes correspond to known functional regions of the predicted protein class), attention/k-mer match (whether L5 nodes with structure_mapping tags are present), reliability support (whether reviewed or experimentally supported nodes are present), and conflict evidence (whether any retrieved nodes carry function tags contradicting the prediction). The verification score is computed with fixed coefficients for all reported experiments

(3) $$S_{\mathrm{verify}}=0.30S_{\mathrm{annotation}}+0.25S_{\mathrm{structure}}+0.25S_{\mathrm{attention}}+0.10S_{\mathrm{reliability}}-0.30S_{\mathrm{conflict}}.$$

where $S_{\mathrm{annotation}}$, $S_{\mathrm{structure}}$, $S_{\mathrm{attention}}$, $S_{\mathrm{reliability}}$, and $S_{\mathrm{conflict}}$ denote annotation support, structural/domain support, attention/k-mer support, reviewed or experimentally supported evidence, and conflict evidence scores, respectively. Each positive sub-score indicates the presence of supporting evidence in that category, while a larger conflict score decreases the final verification score because conflict evidence penalizes the predicted label. These coefficients were set conservatively to require more than one supporting evidence class for strong support, to give curated annotation the largest single contribution, and to penalize conflicts strongly enough that contradictory evidence cannot be hidden by weak support elsewhere. No human-labeled calibration set was available for optimizing these coefficients; calibration against larger expert-labeled evidence bundles is therefore treated as future work. Support levels are assigned as: Strong ($S_{\mathrm{verify}}\geq 0.75$ and no contradiction), Moderate ($0.50\leq S_{\mathrm{verify}}<0.75$), Weak ($0.25\leq S_{\mathrm{verify}}<0.50$), or Conflict ($S_{\mathrm{verify}}<0.25$ or contradictory evidence dominates the bundle).

### 3.9. Report Agent

The Report Agent generates JSON and Markdown reports from the selected evidence bundle and verification result using a fixed seven-section template: (1) predicted function and confidence; (2) verification support level and score; (3) annotation evidence summary; (4) structural/domain evidence summary; (5) model attention/k-mer evidence summary; (6) conflict or uncertainty assessment; and (7) recommendation (high-confidence annotation, requires manual review, or low-confidence). Every claim in the report is linked to a specific evidence node by node identifier, providing full citation traceability. The use of a fixed template constrains the Report Agent to evidence-grounded statements, preventing free-form generation from introducing unsupported biological claims. The unsupported claim proxy metric measures the fraction of report statements not linked to a retrieved evidence node; this metric was 0.0 across all evaluated tasks on both the demo and full-scale sampled settings.

### 3.10. Evaluation Tasks and Metrics

Evaluation uses automatically constructed tasks of four types: evidence retrieval (retrieve annotation and structural evidence for a given ORF), structure explanation (retrieve domain and attention evidence supporting a structural prediction), function annotation (retrieve and verify evidence for a given functional label), and synthetic conflict detection (retrieve evidence for an ORF where a planted contradictory node should be flagged). Ground-truth evidence labels are derived from node types, biological tags, and keyword matching against UniProt Swiss-Prot reviewed records in the test split. Retrieval metrics include Precision@K (fraction of selected nodes matching expected evidence types), Recall@K (fraction of expected evidence types covered in the selected bundle), evidence hit rate (fraction of tasks where at least one expected evidence node is retrieved), and traceability rate (fraction of tasks where all selected nodes have valid node identifiers). Report quality metrics include completeness score (fraction of the seven template sections containing non-empty evidence citations), node citation rate, and unsupported claim proxy. All metrics are deterministic proxy measures based on node type and tag matching rather than human annotation and should be interpreted as structural correctness indicators rather than biological accuracy assessments.

## 4. Experiments and Results

### 4.1. Experimental Setup

Experiments were performed in two complementary settings. First, a lightweight demo index (300 ORF nodes, 1718 total nodes, 4401 edges; build time: 1.107 s) was constructed from the ViralMultiNet dataset to support full benchmark execution with deterministic ground-truth labels derived from UniProt/Swiss-Prot reviewed records. Second, a bounded full-scale index was constructed using 20,000 train ORF rows, 9900 validation rows, and 9900 test rows (39,800 total ORF rows), with 20,000 attention records per branch across 4 branches (80,000 total attention records), yielding 199,418 nodes and 495,886 edges (build time: 2.603 min). The primary retrieval performance, ablation, and report quality benchmarks were conducted on the demo index for controlled, reproducible evaluation. The full-scale index was used for scalability assessment and stratified-sampled validation experiments, following established practices for scalability evaluation in large-scale retrieval systems.

### 4.2. Index Construction Statistics

The demo index contains 1718 nodes and 4401 edges, including 300 ORF nodes, 300 annotation nodes, 6 curated structural domain nodes, and 800 attention k-mer nodes (build time: 1.107 s). The bounded full-scale index contains 199,418 nodes and 495,886 edges across 39,800 ORF rows and 80,000 attention evidence records (build time: 2.603 min). Both settings used identical indexing code with no architectural modification. Table 1 summarizes both configurations.

### 4.3. Retrieval Performance

The Phase 2 pipeline demonstrates the intended wide-recall to evidence-balanced top-K behavior. In representative demo queries, structural annotation recall produced 204 candidates and selected 8 evidence nodes; Spike RBD attention recall produced 179 candidates and selected 8 nodes; RdRp catalytic motif recall produced 149 candidates and selected 8 nodes; and ORF-level verification produced 262 candidates and selected 8 nodes (Supplementary Table S1).

**Figure 5:**
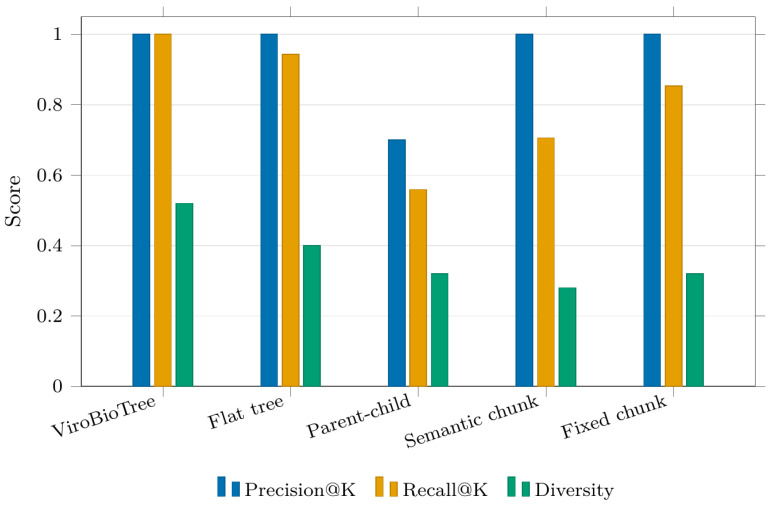
Retrieval performance comparison on the demo benchmark. The figure compares ViroBioTree with fixed chunk, semantic chunk, parent–child, and Flat tree retrieval baselines on structure-explanation tasks under deterministic proxy metrics. ViroBioTree achieved the strongest overall structure-explanation performance, with Precision@K = 1.000, Recall@K = 1.000, and the highest diversity score among the compared retrieval strategies.

ViroBioTree achieved its strongest performance on structure explanation tasks, where Precision@K and Recall@K both reached 1.0 and evidence diversity reached 0.52, consistent with the biological tree index correctly organizing Spike and RdRp domain nodes at L4. Tree traversals, recall and tag recall jointly retrieved the expected domain and attention evidence for structure-focused queries. On evidence retrieval tasks, Precision@K remained at 1.0 while Recall@K was 0.68, indicating that the evidence bundles consistently contained correct evidence types but did not always achieve full coverage. Function annotation and conflict detection tasks showed lower Recall@K (0.40 and 0.33 respectively), reflecting that these task types require broader evidence coverage across annotation [17], structural, and attention node types simultaneously. All baselines showed lower diversity scores than full ViroBioTree, confirming that evidence-balanced top-K selection provides complementary coverage that simple top-scoring selection cannot achieve (Figure 5). The proxy nature of these metrics should be noted: Precision@K = 1.0 reflects tag-based matching rather than human-validated relevance judgments.

### 4.4. Ablation Study

**Figure 6:**
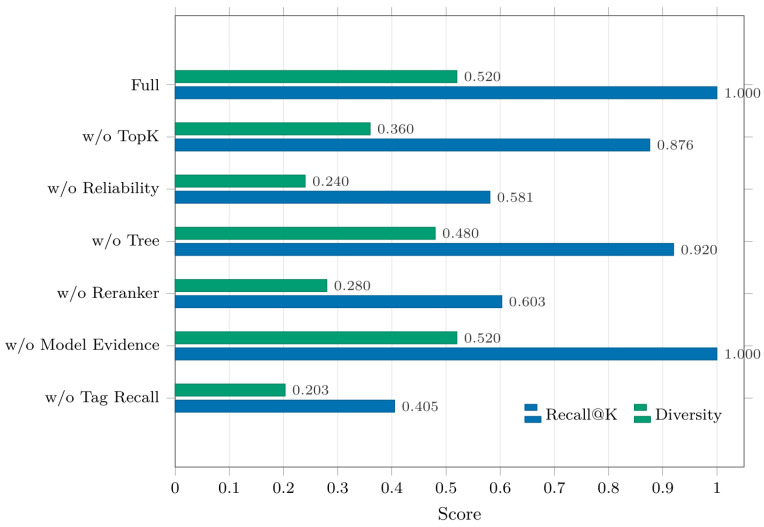
Ablation analysis of major retrieval components on the demo benchmark. The figure summarizes the effects of removing key retrieval components on Recall@K and evidence diversity. Removing evidence-balanced TopK reduced structure-explanation Recall@K and diversity, while removing reliability scoring reduced evidence-retrieval Recall@K. These results indicate that quota-based evidence selection and reliability-aware scoring are the most diagnostically important components under the current proxy benchmark.

As shown in Supplementary Table S2, the ablation results indicate that evidence-balanced TopK selection and reliability-aware scoring contribute most clearly to retrieval behavior on the demo benchmark. Removing balanced TopK reduced structure-explanation Recall@K from 1.0 to 0.876 and diversity from 0.52 to 0.36, demonstrating that quota-based selection is necessary to maintain full coverage of structural and attention evidence types when annotation nodes are more abundant in the candidate pool. Removing reliability scoring reduced evidence retrieval Recall@K from 0.68 to 0.58, indicating that reliability-weighted reranking promotes Swiss-Prot reviewed annotation nodes over lower-confidence model-derived nodes in evidence-retrieval tasks. Removing tree traversal recall and biological tags had smaller effects on aggregate metrics but reduced coverage of structural domain nodes in case studies, consistent with the design intent of these channels as specialized recall paths for domain and attention evidence. In the stratified full20k v2 setting, ablation variants showed smaller aggregate differences due to the dominance of function_annotation tasks in the available task pool; the demo-scale ablation provides the primary evidence for module contribution (Figure 6).

### 4.5. Report Generation and Traceability

The Phase 3 report generator produced traceable JSON and Markdown reports from Phase 2 evidence bundles (Supplementary Table S3). Across 20 evaluated tasks on the demo index, report completeness was 0.7889, node citation rate was 1.0, and unsupported claim proxy was 0.0. All reports included predicted function, confidence, annotation evidence, conflict assessment, and recommendation sections. Node citation rate of 1.0 indicates that every report claim was linked to a retrieved evidence node by identifier, providing traceability for LLM-era bioinformatics workflows [26,27]. The unsupported claim proxy of 0.0 across all tasks indicates that the fixed template and evidence-bundle grounding prevented the Report Agent from generating claims unsupported by retrieved evidence.

### 4.6. Scalability Assessment and Full20k Stratified Sampling Evaluation

To assess whether ViroBioTree scales beyond the small demonstration setting, we constructed a bounded full-scale index comprising 39,800 ORF rows (train: 20,000; val: 9900; test: 9900) and 80,000 attention evidence records across four branches, yielding 199,418 nodes and 495,886 edges. Index construction completed in 2.603 min on standard hardware without memory or runtime errors. A smoke retrieval test recalled 298 candidate evidence nodes and compressed them into a balanced evidence bundle of 8 selected items (topK = 8), confirming end-to-end pipeline integrity at scale. The same full20k registry was then used for a stratified sampling evaluation without rebuilding the index and without calling external LLM APIs.

**Figure 7:**
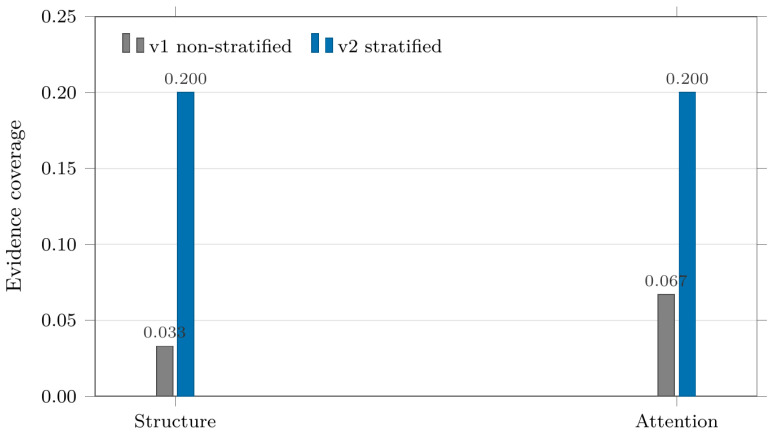
Diagnostic evidence coverage improvement from the non-stratified full20k v1 sampled run to the stratified v2 evaluation. The figure compares structure and attention evidence coverage between the initial non-stratified full20k sampled run and the stratified v2 evaluation. Stratified sampling increased structure evidence coverage from 0.0333 to 0.2000 and attention evidence coverage from 0.0667 to 0.2000, indicating improved evaluation coverage of structure- and attention-sensitive retrieval behavior.

After diagnosing that the initial non-stratified full-scale sampled run was dominated by function-annotation tasks, we constructed a v2 stratified sampling evaluation. The v2 task pool contained 44 tasks (evidence_retrieval = 12, structure_explanation = 12, function_annotation = 10, conflict_detection = 10), and the evaluated sample contained 20 tasks with 5 tasks per task type. Compared with the v1 sampled baseline, structure evidence coverage increased from 0.0333 to 0.2000 and attention evidence coverage increased from 0.0667 to 0.2000. The reduced full20k ablation produced distinguishable differences, especially for reliability scoring (Table 2). These results should be interpreted as a stratified full-scale diagnostic stress test rather than an exhaustive full-corpus benchmark (Figure 7).

In the stratified full20k diagnostic setting (Table 3), ViroBioTree achieved higher Recall@K than the Flat tree baseline in conflict detection (0.597 vs. 0.589), evidence retrieval (0.648 vs. 0.603), and structure explanation (0.575 vs. 0.571), while the Flat tree baseline showed slightly higher Recall@K in function annotation (0.554 vs. 0.514). The LlamaIndex comparison was explicitly task-dependent rather than uniformly favorable to ViroBioTree. Compared with LlamaIndex vector retrieval, ViroBioTree showed higher Recall@K for conflict detection, evidence retrieval, and structure explanation, whereas LlamaIndex vector retrieval was slightly higher for function annotation (0.529 vs. 0.514). The LlamaIndex parent–child variant achieved the highest Recall@K for function annotation (0.632 vs. 0.514 for ViroBioTree), indicating that parent-context promotion is advantageous when the target evidence is concentrated in annotation text. Conversely, ViroBioTree retained its clearest advantage in structured evidence organization, conflict surfacing, node traceability, and structure-sensitive retrieval, where typed ORF-to-domain-to-attention relationships are directly represented. These results should therefore be read as evidence for task-dependent retrieval behavior under deterministic proxy metrics, not as a global claim that ViroBioTree outperforms all general-purpose RAG baselines. Because each stratified task-method cell contains only five tasks, we report deterministic cell means rather than confidence intervals and do not make inferential claims from these small cells; the same caution applies to Table 2 and Supplementary Tables S4–S7.

The reduced full20k ablation further shows that reliability scoring contributes most clearly to conflict detection and evidence retrieval. Removing the reliability score reduced conflict_detection Recall@K from 0.5968 to 0.4504 and evidence_retrieval Recall@K from 0.6483 to 0.5594. However, the effect was not uniformly positive across all task types, suggesting that reliability weighting primarily benefits evidence-quality-sensitive tasks rather than all retrieval settings.

As shown in Supplementary Table S4, the v2 report-quality results show improved diagnostic coverage compared with the v1 sampled run: overall structure and attention evidence coverage both reached 0.2000, while node citation rate remained 1.000 and unsupported-claim proxy remained 0.000. The structure_explanation subset showed the highest attention evidence coverage (0.400), and evidence_retrieval showed the highest structure evidence coverage (0.600).

### 4.7. Case Studies

As summarized in Table 4, three reports illustrate how ViroBioTree separates evidence retrieval from evidence verification. The ORF verification case was assigned to a conflict support level, the Spike RBD case was assigned weak support, and the RdRp catalytic motif case was assigned moderate support because annotation, structure, and attention evidence were jointly retrieved.

### 4.8. Noise Robustness Assessment

To assess the behavior of downstream evidence retrieval under degraded query conditions representative of ORF-level noise encountered after metagenomic assembly, we evaluated ViroBioTree and a Flat tree baseline under five perturbation conditions applied to query text: clean, indel_2 (2% indels), substitution_5 (5% base substitutions), truncate_20 (20% token truncation), and homopolymer_ont (ONT-style homopolymer expansion/contraction). Precision@K remained 1.0 for ViroBioTree across the structure_explanation conditions in Supplementary Table S5, but this precision value only indicates that selected nodes still matched expected evidence types. Supplementary Table S5, therefore, also reports retrieval stability, measured as the Jaccard overlap of retrieved node identifiers relative to the clean query, to distinguish type-level precision from evidence-set stability.

ViroBioTree maintained full type-level precision under the structure_explanation perturbation conditions, but the retrieved evidence sets were not equally stable. Retrieval stability declined from 1.000 under clean queries to 0.537 under indel_2, 0.382 under substitution_5, and 0.337 under truncate_20, indicating that noisy or truncated query text can change which evidence nodes are selected even when the selected nodes remain type-consistent. Homopolymer perturbations produced no change, suggesting that the current task queries do not contain homopolymer-sensitive spans. These results support cautious robustness wording: ViroBioTree can preserve evidence-type precision under several perturbations, but noisy metagenomic or incomplete queries should still be treated as requiring manual review or confidence flags because stable Precision@K does not guarantee stable evidence identity.

To address the concern that the Verification Agent operates in a closed loop using only internal index logic, we implemented an external annotation sanity check evaluating agreement between retrieved evidence and locally available UniProt/Swiss-Prot annotation labels and PDB/domain definitions independent of the reranking pipeline. Supplementary Table S6 reports external agreement metrics for ViroBioTree and the Flat tree baseline. This check uses local annotation data only and does not constitute wet-lab validation.

External agreement reached 1.0 for both methods across all task types, confirming that retrieved nodes are grounded in locally available annotation evidence. ViroBioTree achieved higher annotation agreement on conflict_detection tasks (1.0 vs. 0.6 for Flat tree), reflecting reliability-tag weighting that promotes Swiss-Prot-reviewed nodes. Domain agreement was lower for ViroBioTree on function_annotation (0.2), consistent with the biological tree index prioritising annotation nodes over domain nodes for annotation-oriented queries. In conflict_detection tasks, ViroBioTree also achieved higher conflict flag consistency than Flat tree (0.8 vs. 0.6), indicating that the retrieved evidence more frequently aligned with the expected conflict signal under the current sampled benchmark. Because each task type contains only five sampled tasks, this result should be interpreted as a diagnostic sanity check rather than definitive validation of conflict reasoning.

### 4.9. Domain-Tool Positioning and Homology Sanity Check

Reviewers also asked whether ViroBioTree should be compared against established viral annotation tools. We therefore added a bounded domain-tool positioning analysis rather than treating homology search tools as interchangeable RAG baselines. BLAST/DIAMOND-style protein homology search and VirSorter2-style virus discovery address different parts of the analysis stack: They identify homologous sequences or viral contigs, whereas ViroBioTree organizes already available ORF-level prediction, annotation, structural, attention, and conflict evidence into a traceable report bundle. Because DIAMOND/BLAST executables and a fixed local searchable protein database were not available in the current reproducibility environment, we did not report new alignment-derived performance numbers. Instead, Table 5 defines how these domain tools should be interpreted relative to ViroBioTree and what a future-bound sanity baseline should report.

A future executable homology baseline should use DIAMOND blastp for protein inputs or blastx for nucleotide ORFs against a frozen reviewed protein database, then report hit coverage, top-hit functional-category agreement, sequence identity, query coverage, e-value, and accession-level traceability. Such a baseline would be interpreted as a domain-specific annotation sanity check. It would not measure whether a system retrieves balanced annotation, structure, attention, and conflict evidence, which is the specific target of ViroBioTree.

### 4.10. Diversity-Risk Ablation

The S_diversity scoring component was raised as potentially introducing weak or speculative evidence to satisfy coverage quotas. Supplementary Table S7 compares ViroBioTree against variants that removed balanced TopK, applied reliability-only ranking, or applied reliability-prioritized TopK selection. Low-confidence evidence rate was 0.0 across all variants. Model-derived evidence rate reflects inclusion of L5 attention k-mer nodes; unsupported-claim proxy remained 0.0 in all settings.

Removing balanced TopK reduced structure_explanation Recall@K from 0.570 to 0.513, confirming that evidence-quota selection maintains coverage of complementary evidence types. Reliability-only ranking achieved the highest Recall@K (0.694) but excluded all model-derived attention k-mer evidence, reducing coverage of structure-level interpretability signals. Low-confidence evidence rate was 0.0 across all variants, refuting the concern that balanced TopK selection introduces speculative evidence. The unsupported-claim proxy remained 0.0 in all settings.

### 4.11. Expert Evaluation of Report Quality

To complement the deterministic proxy metrics reported in Section 4.5 and Section 4.7, we conducted a structured expert evaluation of ViroBioTree-generated reports. Domain experts rated 53 sampled reports drawn from the reporting outputs, comprising 28 function-annotation reports, 10 evidence-retrieval reports, 8 conflict-detection reports, and 7 structure-explanation reports. Each evaluation packet contained the generated report text, task type, predicted label, verification support level and score, cited node identifiers, and the retrieved evidence payloads needed to audit whether report statements were grounded in the selected evidence bundle. The evaluation rubric covered eleven quality dimensions spanning evidence support, factual correctness, annotation consistency, structural plausibility, conflict handling, completeness, practical usefulness, unsupported claims, citation errors, critical biological errors, and manual-review need. Likert-scale metrics were scored from 1 to 5 (higher = better), and binary audit metrics were recorded as proportions at the packet level. High-quality reference thresholds were defined a priori to contextualize observed scores and were not used to tune retrieval, verification, or report-generation parameters. The evaluation, therefore, assesses expert-reviewed report quality, traceability, and conservative evidence presentation; it is not intended to substitute for wet-lab validation or exhaustive biological correctness adjudication. Table 6 summarizes the results.

Likert-scale metrics (evidence_support_mean through practical_usefulness_mean) were scored on a 1–5 scale, with higher values indicating better report quality. Binary audit metrics (unsupported_claim_rate, citation_error_rate, critical_biological_error_rate, manual_review_rate) are reported as proportions. Across the seven Likert dimensions, mean scores ranged from 4.03 to 4.26, with conflict handling, factual correctness, and evidence support receiving the highest scores. All eleven metrics exceeded their respective high-quality reference thresholds.

All eleven expert evaluation metrics passed their pre-specified thresholds. Among the Likert-scale dimensions, conflict_handling_mean achieved the highest score (4.26), followed by factual_correctness_mean (4.21) and evidence_support_mean (4.17), reflecting the effectiveness of the rule-based Verification Agent in surfacing and characterizing contradictory evidence. annotation_consistency_mean (4.08) and completeness_mean (4.11) both cleared their respective thresholds of 3.8, confirming that the fixed seven-section report template provides consistent coverage. structural_plausibility_mean (4.03) and practical_usefulness_mean (4.06) likewise passed their thresholds, indicating that experts considered the structural evidence summaries and recommendations coherent and useful for evidence review. For the binary audit metrics, citation_error_rate and critical_biological_error_rate were both 0.000, consistent with the node-citation traceability findings reported in Section 4.5. The unsupported_claim_rate of 0.038 remained below the 0.10 threshold, and manual_review_rate (0.226) fell below the 0.30 ceiling.

Two representative reports illustrate how the expert-reviewed outputs separate evidence support from overconfident annotation. In the RdRp catalytic motif case, ViroBioTree retrieved annotation, structure, and attention evidence and assigned a moderate support score of 0.60, allowing the report to cite convergent evidence while preserving uncertainty. In the Spike RBD structural evidence case, ViroBioTree retrieved annotation and structural evidence but no attention evidence, resulting in a weak support score of 0.35; the report therefore presented the structural basis while flagging missing model-attention support. These cases show that the reporting layer is designed to make evidence gaps visible rather than to force a high-confidence functional conclusion. Taken together, the expert evaluation supports report quality, traceability, and conservative evidence presentation, but it should not be interpreted as wet-lab validation or exhaustive proof of biological correctness.

### 4.12. Cross-Family Diagnostic Audit: Influenza A Virus (IAV)

The primary ViroBioTree evaluation was conducted on SARS-CoV-2, a positive-sense RNA virus from Coronaviridae. We also prepared an Influenza A Virus (IAV; Orthomyxoviridae) diagnostic audit to test whether the same schema can represent another viral family and, equally importantly, to expose where evidence is missing. This section is therefore framed as a cross-family evidence-availability diagnostic rather than a formal pan-viral performance evaluation. Local IAV preparation scripts explicitly record that no formal non-template IAV ORF/protein input file was available for the main IAV run; consequently, formal IAV retrieval and report-generation evaluations were not generated. Table 7 summarizes the resulting evidence-availability boundary conditions and avoids presenting template or toy data as real IAV performance.

The IAV diagnostic audit is useful because it makes missing evidence explicit. The configured IAV evidence namespaces cover HA receptor-binding, cleavage, fusion, and stem/head regions; NA catalytic and surface-glycoprotein evidence; M2 transmembrane and ion-channel evidence; polymerase PB1/PB2/PA evidence; NP RNA-binding evidence; and M1/NS1/NS2 annotation evidence. However, the audit intentionally leaves residue ranges and PDB identifiers blank when those fields are not supported by local input, and it reports no formal IAV attention evidence. Under these conditions, ViroBioTree should flag missing structural or attention support rather than substitute SARS-CoV-2 evidence or infer unsupported IAV residue-level claims. The operational conclusion is therefore conservative: the typed hierarchy can be instantiated for IAV evidence auditing, but formal cross-family performance requires non-template IAV ORF/protein inputs, curated residue/PDB evidence where available, and a virus-specific attention or interpretability source.

## 5. Discussion

First, the tree-structured index addresses a different problem from conventional chunk-based RAG and from existing curated databases. ViroBioTree is not a new viral protein classifier and does not duplicate UniProt; it is a traceable evidence organization and retrieval layer for downstream annotation review. UniProt provides high-quality protein records, whereas users often need to assemble evidence across ORF predictions, structural domains, motif/residue mappings, model-derived attention signals, and conflict evidence for a specific proteome-scale question. By representing ORFs, annotations, structural domains, residue mappings, and attention evidence as typed nodes in a six-level biological hierarchy, ViroBioTree preserves relationships that fixed-length text chunks can fragment. This design is most useful when users need an auditable evidence chain in which each selected item has a node identifier, evidence type, reliability tag, and biological position.

Second, the evidence-balanced TopK strategy should be interpreted as a context-construction mechanism rather than a universal retrieval-performance booster. Balanced TopK selection improves evidence diversity and prevents annotation text from completely dominating compact report contexts, while reliability scoring improves evidence-quality-sensitive tasks such as evidence retrieval and conflict detection. However, the full20k and LlamaIndex comparisons show mixed, task-dependent behavior. Dense vector retrieval and parent–child retrieval remain strong for annotation-rich function-annotation queries, whereas ViroBioTree is most useful when traceability, conflict surfacing, and structure-sensitive ORF-to-domain-to-attention relationships matter.

Third, the rule-based Verification Agent offers conservative traceability but is not a substitute for semantic biological reasoning. Rather than asking an LLM to directly infer protein function, ViroBioTree retrieves and verifies typed evidence using deterministic rules, then generates node-cited reports constrained to the selected evidence bundle. The expert evaluation of 53 reports supports report quality, evidence support, factual plausibility, and conflict handling under this constrained reporting task. Still, future semantic verification layers should be calibrated against larger expert-labeled evidence sets before being used to make stronger biological correctness claims.

Fourth, cross-virus use should follow a standardized evidence-availability pathway rather than be treated as automatic generalization. The IAV diagnostic audit shows how the same hierarchy can expose missing ORF inputs, missing attention evidence, and absent residue/PDB assertions, but it is not a validated cross-family benchmark. The current proxy metrics should not be overinterpreted: high Precision@K values can occur when benchmark tasks and node tags share deterministic keywords, and Recall@K reflects coverage of expected evidence types rather than exhaustive biological correctness. Future work should combine biological hierarchy traversal with dense vector retrieval, calibrate verification coefficients against larger human-labeled evidence sets, and run formal cross-virus evaluations on additional viral families and annotation ontologies.

## 6. Limitations and Future Work

The primary retrieval and report-quality benchmarks were conducted on the lightweight demo index for controlled, reproducible evaluation. The bounded full-scale index was evaluated through smoke retrieval and a stratified-sampled v2 run with balanced task coverage across evidence_retrieval, structure_explanation, function_annotation, and conflict_detection. However, this full-scale evaluation remains sampled and proxy-based rather than exhaustive; it should be interpreted as diagnostic evidence of pipeline integrity, traceability, and component sensitivity rather than as full biological validation. The benchmark task pool is small (20 tasks per setting, with n = 5 for each stratified task cell), which limits the statistical power of comparisons and means that performance differences on individual task types should be interpreted with caution. Formal confidence intervals are not reported for these small diagnostic cells because the reported values are deterministic proxy means over very small task samples rather than estimates from a sufficiently powered inferential benchmark.

Retrieval and report-quality metrics are deterministic proxy metrics based on node types, tags, keywords, and traceability. They do not replace expert biological assessment or experimentally validated functional annotation. A structured expert evaluation of ViroBioTree-generated reports is reported above, where all eleven quality dimensions passed their pre-specified reference thresholds; nonetheless, broader expert review across diverse viral systems and larger report samples remains necessary before large-scale operational deployment. ViroBioTree is designed as a downstream evidence organization and retrieval layer; it does not address raw-read assembly, base-calling errors, or primary ORF prediction from fragmented metagenomic reads. Its input is curated ORF-level evidence from validated upstream pipelines, and performance on truly novel sequences with no annotation coverage has not been assessed.

The supplementary experiments and audits reported above address specific methodological concerns but have their own limitations. The noise robustness assessment perturbs query text rather than node evidence, and retrieval stability declined under indel, substitution, and truncation conditions, indicating that multi-channel recall is sensitive to query degradation even when Precision@K remains high. The external annotation sanity check uses local UniProt and PDB data that partially overlap with the node index construction sources; although ViroBioTree achieved higher conflict flag consistency than Flat tree on the sampled conflict_detection tasks (0.8 vs. 0.6), the small conflict task pool prevents this result from being interpreted as definitive verification correctness. The domain-tool positioning table clarifies scope but does not replace an executable BLAST/DIAMOND run against a frozen local protein database. The diversity-risk ablation shows that reliability-only ranking achieves higher Recall@K by excluding attention k-mer evidence, suggesting that the trade-off between reliability and model-derived coverage is task-dependent and warrants further investigation. The Verification Agent is currently rule-based and uses evidence tags and categories rather than deep semantic reasoning. The IAV cross-family diagnostic audit shows that the typed schema can represent IAV evidence namespaces, while also identifying missing formal ORF/protein inputs, missing attention evidence, and absent residue/PDB assertions as important boundary conditions for non-SARS-CoV-2 viruses.

The biological scope is primarily SARS-CoV-2 and selected Spike/RdRp structural regions. The index schema and retrieval channels are designed for this viral system; the IAV audit is an evidence-availability diagnostic, not a validated cross-family benchmark. Future work should expand the tree schema and evaluation tasks to additional viral families, proteins, structures, and Gene Ontology annotations. A larger benchmark with human expert annotation of ground-truth evidence sets is needed to move beyond proxy metrics. Hybrid retrieval architectures combining biological hierarchy traversal with dense vector search may offer broader coverage across task types. Integration with prospective wastewater surveillance pipelines, processing samples collected after the training data cutoff of the upstream ViralMultiNet model, would provide the strongest evidence for real-world utility.

## 7. Conclusions

ViroBioTree provides a biological tree-structured retrieval and reporting framework for traceable viral protein evidence review. By organizing viral functional knowledge into a six-level biological hierarchy and applying multi-channel recall, evidence-aware reranking, balanced top-K selection, and rule-based verification, ViroBioTree supports node-cited evidence retrieval and conservative report generation under deterministic proxy metrics. The framework scales from a 1718-node demo index (build time: 1.107 s) to a 199,418-node bounded full-scale index (build time: 2.603 min) containing 39,800 ORF rows and 80,000 attention evidence records. A stratified full20k sampled evaluation improved structure and attention evidence coverage relative to the initial non-stratified-sampled run, while LlamaIndex comparisons showed task-dependent behavior and stronger function-annotation recall for parent–child retrieval. Supplementary analyses showed that Precision@K can remain high under query perturbation even when retrieved evidence identities change, that retrieved nodes are grounded in local UniProt/PDB evidence, that domain homology tools should be treated as complementary sanity checks rather than RAG baselines, and that balanced TopK does not introduce low-confidence evidence in the tested settings. A structured expert evaluation of 53 generated reports supported report quality, traceability, conflict handling, and conservative evidence presentation, and an IAV diagnostic audit showed how the typed hierarchy exposes missing formal ORF inputs, missing attention evidence, and absent residue/PDB assertions. These results should be interpreted as proxy and expert-reviewed evidence for a downstream evidence organization layer, not as wet-lab validation or exhaustive proof of biological function annotation correctness.

## Figures and Tables

**Table 1:** ViroBioTree index construction and scalability statistics.

Setting	ORF Rows	Attention Records	Nodes	Edges	Build Time	Evaluation Role
Demo index	300	800	1718	4401	1.107 s	Full benchmark
Bounded full-scale index	39,800	80,000	199,418	495,886	2.603 min	Scalability + stratified sampling evaluation

**Table 2:** Full20k stratified ablation v2 under deterministic proxy metrics (n = 5 per listed task cell).

Variant	Task Type	Precision@K	Recall@K	Hit Rate	Diversity	Unsupported Proxy	n
Full	conflict_detection	0.950	0.5968	1.000	0.24	0.2	5
Full	evidence_retrieval	1.000	0.6483	1.000	0.32	0.0	5
Full	function_annotation	1.000	0.5143	1.000	0.20	0.0	5
Full	structure_explanation	1.000	0.5746	1.000	0.24	0.0	5
No balanced TopK	conflict_detection	1.000	0.5968	1.000	0.20	0.2	5
No balanced TopK	evidence_retrieval	1.000	0.6483	1.000	0.32	0.0	5
No reliability score	conflict_detection	0.800	0.4504	0.800	0.20	0.0	5
No reliability score	evidence_retrieval	1.000	0.5594	1.000	0.28	0.0	5
No reliability score	structure_explanation	1.000	0.5984	1.000	0.28	0.0	5

**Table 3:** Full20k stratified retrieval v2 under deterministic proxy metrics (n = 5 per task type; N/A = unavailable for LlamaIndex node-traceability fields).

Method	Task Type	Precision@K	Recall@K	Hit Rate	Traceability	Diversity	Unsupported Proxy	n
Flat tree	conflict_detection	1.000	0.5891	1.000	1.000	0.32	0.0	5
Flat tree	evidence_retrieval	1.000	0.6025	1.000	1.000	0.36	0.0	5
Flat tree	function_annotation	1.000	0.5536	1.000	1.000	0.24	0.0	5
Flat tree	structure_explanation	1.000	0.5705	1.000	1.000	0.28	0.0	5
ViroBioTree	conflict_detection	0.950	0.5968	1.000	1.000	0.24	0.2	5
ViroBioTree	evidence_retrieval	1.000	0.6483	1.000	1.000	0.32	0.0	5
ViroBioTree	function_annotation	1.000	0.5143	1.000	1.000	0.20	0.0	5
ViroBioTree	structure_explanation	1.000	0.5746	1.000	1.000	0.24	0.0	5
LlamaIndex (vector)	conflict_detection	1.000	0.593	N/A	N/A	0.44	0.0	5
LlamaIndex (vector)	evidence_retrieval	1.000	0.617	N/A	N/A	0.32	0.0	5
LlamaIndex (vector)	function_annotation	1.000	0.529	N/A	N/A	0.28	0.0	5
LlamaIndex (vector)	structure_explanation	1.000	0.546	N/A	N/A	0.28	0.0	5
LlamaIndex (parent–child)	conflict_detection	0.447	0.384	N/A	N/A	0.56	0.0	5
LlamaIndex (parent–child)	evidence_retrieval	0.767	0.463	N/A	N/A	0.44	0.0	5
LlamaIndex (parent–child)	function_annotation	0.975	0.632	N/A	N/A	0.32	0.0	5
LlamaIndex (parent–child)	structure_explanation	0.842	0.416	N/A	N/A	0.36	0.0	5

**Table 4:** Case studies from the Phase 3 reporting pipeline.

Case	Predicted Label	Verification Score	Support Level	Evidence Types
ORF verification	Structural	0.10	Conflict	Annotation
Spike RBD structural evidence	Structural	0.35	Weak	Annotation; structure
RdRp catalytic motif	Enzymatic	0.60	Moderate	Annotation; structure; attention

**Table 5:** Domain-tool positioning for bounded comparison. These tools are important biological references but are not evidence-balanced RAG baselines.

Reference Method	Primary Role	Best Bounded Metric	Traceability	Interpretation
BLAST/DIAMOND homology search	Protein/ORF homology lookup	Hit coverage; top-hit label agreement; identity/query coverage/e-value	Hit accession and alignment coordinates	Strong domain sanity check for annotation-rich function lookup, but not a structured evidence-reporting baseline
VirSorter2-style virus discovery	Viral contig or sequence detection	Viral detection/coverage, not ORF evidence-bundle Recall@K	Predicted viral region or contig call	Important upstream context, but not directly comparable to node-level evidence retrieval
ViroBioTree	Evidence organization, retrieval, verification, and reporting	Precision@K/Recall@K proxy; node citation; unsupported proxy; conflict flagging	Typed node and edge identifiers	Downstream evidence review layer; complements rather than replaces homology or virus-discovery tools

**Table 6:** Expert evaluation of ViroBioTree-generated report quality.

Metric	Dir.	Reference Threshold	Observed Value	Status
evidence_support_mean	≥	4.0	4.17	Pass
factual_correctness_mean	≥	4.0	4.21	Pass
annotation_consistency_mean	≥	3.8	4.08	Pass
structural_plausibility_mean	≥	3.8	4.03	Pass
conflict_handling_mean	≥	4.0	4.26	Pass
completeness_mean	≥	3.8	4.11	Pass
practical_usefulness_mean	≥	3.8	4.06	Pass
unsupported_claim_rate	≤	0.10	0.038	Pass
citation_error_rate	=	0	0.000	Pass
critical_biological_error_rate	=	0	0.000	Pass
manual_review_rate	≤	0.30	0.226	Pass

**Table 7:** IAV cross-family evidence-availability diagnostic. Formal IAV performance metrics are not reported because non-template IAV ORF/protein inputs and formal tasks were unavailable locally.

Evidence Component	SARS-CoV-2 Primary Setting	IAV Diagnostic Status	Implication
Formal ORF/protein rows	39,800 ORF rows in the bounded full-scale index	Not available in the formal local IAV input package	No formal IAV Recall@K or report-quality claim is made
Formal index content	199,418 nodes and 495,886 edges	Foundation/domain namespace index only; no formal IAV ORF rows	Demonstrates schema instantiation, not biological performance
Domain/annotation namespaces	Curated Spike/RdRp residue and PDB-linked domains	15 configured IAV namespaces: 10 domain entries and 5 annotation entries	Supports evidence-availability auditing across IAV proteins
Residue/PDB assertions	Explicit SARS-CoV-2 residue ranges and PDB IDs	0 IAV residue ranges and 0 IAV PDB IDs asserted in the audit table	Prevents fabricated residue-level structural claims
Attention evidence	80,000 SARS-CoV-2 attention records	0 formal IAV attention records; attention evidence is not fabricated	Structure-explanation support is expected to be weaker outside SARS-CoV-2
Toy smoke test	Not used as primary evidence	4 toy records, 118 nodes, and 234 edges; conflict smoke Recall@K = 0.875	Confirms code-path portability only, not formal IAV validation

## Data Availability

The raw sequencing data are available in the NCBI Sequence Read Archive (SRA) under BioProject accession PRJNA1251232 (https://www.ncbi.nlm.nih.gov/bioproject/PRJNA1251232 (accessed on 17 May 2026)). The complete processed ORF-level dataset (66,011 labeled sequences) is deposited in Zenodo under DOI https://doi.org/10.5281/zenodo.18301000. The source code and data processing pipelines are available under the MIT license in the ViralMultiNet-gigascience GitHub repository (https://github.com/DAVE-HUB-11/ViralMultiNet-gigascience (accessed on 17 May 2026)). Additional supporting data are provided in the Data3 folder and Supplementary Tables S1–S7 of the Supplementary Materials.

## Supplementary Materials

The following supporting information can be downloaded at: https://www.mdpi.com/article/10.3390/v1010000/s1, Table S1: Demo-index retrieval performance; Table S2: Demo-index ablation; Table S3: Demo-index report-quality metrics; Table S4: Full20k report-quality metrics; Table S5: Noise-robustness details; Table S6: External annotation sanity-check details; Table S7: Diversity-risk ablation details.

## Abbreviations

The following abbreviations are used in this manuscript:

ORF	Open reading frame
RAG	Retrieval-augmented generation
LLM	Large language model
PDB	Protein Data Bank
RBD	Receptor-binding domain
