# Supplementary material for: ViroBioTree: A Tree-Structured Biological Evidence Retrieval Framework for Viral Protein Function Annotation
Source: Viruses. 2026 Jun 9;18(6):656. doi: 10.3390/v18060656 (PMC13307837; doi:10.3390/v18060656)
Supplement: Supplementary file 1 [file viruses-18-00656-s001.zip › viruses-4354331-supplementary.pdf]

Supplementary Tables for ViroBioTree Manuscript

These supplementary tables preserve detailed supporting results moved out of the main manuscript to keep the primary text focused on core comparisons, representative cases, expert evaluation, and the cross-family diagnostic audit.

Table 1: Supplementary Table S1. Retrieval performance comparison under deterministic proxy metrics (demo index).

**Method**	**Task type**	**Precision@K**	**Recall@K**	**Diversity**	**Unsupported proxy**
ViroBioTree	structure_explanation	1.000	1.000	0.52	0.0
ViroBioTree	evidence_retrieval	1.000	0.6807	0.28	0.0
Flat tree	structure_explanation	1.000	0.9429	0.40	0.0
Parent-child	structure_explanation	0.700	0.5591	0.32	0.0
Semantic chunk	structure_explanation	1.000	0.7057	0.28	0.0
Fixed chunk	structure_explanation	1.000	0.8533	0.32	0.0

Table 2: Supplementary Table S2. Ablation study results under deterministic proxy metrics on the demo index.

**Variant**	**Main affected task**	**Precision@K**	**Recall@K**	**Diversity**	**Main observation**
Full	structure_explanation	1.000	1.000	0.52	Best structural evidence coverage
No balanced TopK	structure_explanation	1.000	0.8762	0.36	Lower recall and evidence diversity
No reliability score	evidence_retrieval	1.000	0.5807	0.24	Lower evidence recall
No tree traversal	structure_explanation	1.000	0.9200	0.48	Reduced structural recall
No reranker	evidence_retrieval	0.850	0.6031	0.28	Lower evidence retrieval precision
No model evidence recall	structure_explanation	1.000	1.000	0.52	No measurable effect in current benchmark
No tag recall	function_annotation	1.000	0.4053	0.2026	Minimal effects under current task design

Table 3: Supplementary Table S3. Report quality evaluation (demo index, 20 tasks).

**Task type**	**Completeness**	**Node citation rate**	**Unsupported proxy**	**Tasks**
All	0.7889	1.000	0.0	20
Evidence retrieval	0.8222	1.000	0.0	5
Function annotation	0.7778	1.000	0.0	12
Conflict detection	0.7778	1.000	0.0	3

Table 4: Supplementary Table S4. Full20k stratified report quality v2 (n = 5 per task type).

Task type	Annotation coverage	Structure coverage	Attention coverage	Citation rate	Unsupported proxy	Completeness	n
All	0.800	0.200	0.200	1.000	0.000	0.8000	20
conflict_detection	0.800	0.000	0.200	1.000	0.000	0.7778	5
evidence_retrieval	1.000	0.600	0.000	1.000	0.000	0.8445	5
function_annotation	0.800	0.000	0.200	1.000	0.000	0.7778	5
structure_explanation	0.600	0.200	0.400	1.000	0.000	0.8000	5

Table 5: Supplementary Table S5. Noise robustness on structure_explanation tasks. Precision@K remained 1.0 for ViroBioTree in all listed conditions, but retrieval stability measures whether the same evidence-node identifiers were recovered relative to the clean query (n = 5 per cell).

Method	Noise Condition	Recall@K	Retrieval Stability
ViroBioTree	clean	0.570	1.000
ViroBioTree	indel_2	0.681	0.537
ViroBioTree	substitution_5	0.710	0.382
ViroBioTree	truncate_20	0.586	0.337
ViroBioTree	homopolymer_ont	0.570	1.000
Flat tree	clean	0.591	1.000
Flat tree	indel_2	0.624	0.846
Flat tree	substitution_5	0.624	0.846
Flat tree	truncate_20	0.591	0.667
Flat tree	homopolymer_ont	0.591	1.000

Table 6: Supplementary Table S6. External annotation sanity check (n = 5 per cell; N/A = not applicable for non-conflict tasks).

Method	Task type	External agreement	Annotation agreement	Domain agreement	Conflict consistency
ViroBioTree	conflict_detection	1.0	1.0	0.2	0.8
ViroBioTree	evidence_retrieval	1.0	0.8	0.6	N/A
ViroBioTree	function_annotation	1.0	0.8	0.2	N/A
ViroBioTree	structure_explanation	1.0	0.6	0.6	N/A
Flat tree	conflict_detection	1.0	0.6	0.8	0.6
Flat tree	evidence_retrieval	1.0	0.8	0.6	N/A
Flat tree	function_annotation	1.0	0.8	0.6	N/A
Flat tree	structure_explanation	1.0	0.8	0.4	N/A

Table 7: Supplementary Table S7. Diversity-risk ablation on structure_explanation and evidence_retrieval tasks (n = 5 per cell).

**Variant**	**Task type**	**Recall@K**	**Diversity**	**Model-derived rate**	**Unsupported proxy**
ViroBioTree	structure_explanation	0.570	0.28	0.450	0.0
No balanced TopK	structure_explanation	0.513	0.24	0.400	0.0
Reliability-prioritized TopK	structure_explanation	0.513	0.24	0.400	0.0
Reliability-only ranking	structure_explanation	0.694	0.40	0.000	0.0
ViroBioTree	evidence_retrieval	0.648	0.32	0.125	0.0
No balanced TopK	evidence_retrieval	0.648	0.32	0.125	0.0
Reliability-only ranking	evidence_retrieval	0.696	0.36	0.000	0.0
